# Diagnostic laparoscopy to investigate unexplained lactic acidosis in critically ill patients - A descriptive single centre cohort study

**DOI:** 10.1016/j.amsu.2018.11.008

**Published:** 2018-11-13

**Authors:** Mohammed Ahmed Sajid, Khurram Shahzad Khan, Zulfiqar Hanif

**Affiliations:** Department of Surgery, University Hospital Hairmyres, NHS Lanarkshire, East Kilbride, G75 8RG, Scotland, UK

**Keywords:** Critically ill, Lactic acidosis, Diagnostic laparoscopy, Emergency exploratory laparotomy, Intensive care unit

## Abstract

**Introduction:**

Unexplained lactic acidosis (LA) in a critically ill patient often prompts investigations to rule out any reversible intra-abdominal cause. Equivocal results can lead to an emergency laparotomy (EL) with subsequent high morbidity and mortality rates. Our objective was to determine the clinical impact of urgent diagnostic laparoscopy (UDL) in such patients.

**Methods:**

This was a descriptive single-centre cohort study. UDL on 28 consecutive critically ill patients with unexplained LA who were referred to a single surgeon over 16 years period were analysed. UDL was proformed either at bedside or in theatre without prior computerised tomography (CT) scan. Patient's demographics, ASA grade, referral route and intraoperative findings were analysed.

**Results:**

Eighteen patients underwent bedside UDL in the critical care setting and further 10 had UDL in theatre. Fourteen patients had normal UDL, out of these 10 had LA secondary to low cardiac output states. Fourteen patients had positive UDL findings. Seven patients had features of mesenteric ischaemia, two had gangrenous gallbladder, two had hepatic ischaemia, one patient had acute pancreatitis, one had gangrenous uterus and one had gastric volvulus. Five of the 14 patients with positive UDL were converted to laparotomy for definitive management. In total, of the 28 patients in the cohort, 23 patients avoided EL.

**Conclusion:**

UDL is useful and feasible investigation for unexplained LA in the critically ill patients and it can avoid unnecessary EL in many patients. We would recommend the use of UDL as a safe and feasible investigation in such patients.

## Highlights

• Lactic Acidosis likely from an intra-abdominal source usually prompts explorative Laparotomy.
• We performed diagnostic laparoscopy for 28 critically ill patients with Lactic Acidosis.
• These patients underwent diagnostic laparoscopy prior to any CT scanning, in either bedside or theatre setting.
• 50% of patients had a normal Laparoscopy results and Explorative laparotomy was prevented in 18% of patients.
• Diagnostic Laparoscopy is safe and feasible when investigating lactic acidosis from an intra-abdominal source and risks are reduced with explorative laparotomy.

## 1 Introduction

Lactic acidosis (LA) defined as a serum lactate of ≥4 mmol/L is a common finding in critically ill patients [1]. It is an indicator of higher morbidity and mortality especially in patients who are relatively unstable as being hightlighted in Surviving Sepsis Campaign Bundle: 2018 update [2]. Lactic acidosis is thought to arise because of a hypoxic environment, where cells either receive an inadequate supply of oxygen or are unable to utilise available oxygen for aerobic respiration. The net effect is an increase in anaerobic respiration, which causes surplus production of lactate. Accumulation of lactate results in lactic acidaemia, and this is commonly seen in critically ill patients due to reduced hepatic and renal clearance [3,4]. Causes such as hypovolaemia and septic shock (e.g. intra-abdominal pathology) cause impaired oxygen delivery to tissues [5]. The end result is a critically ill patient with potentially reversible LA.

Diagnosing the intra-abdominal cause of LA in critically ill patients remains challenging. Patients are usually sedated, intubated and unstable and are commonly too unwell to undergo radiological investigations like computed tomography (CT) scan. In such cases, suspicion of an intra-abdominal catastrophe often results in an emergency laparotomy (EL) [[6], [7], [8]] which carries its own morbidity and mortality [9,10].

We propose bedside UDL as a useful diagnostic tool for the investigation of intra-abdominal cause of LA in critically ill patients where medical causes of LA have been excluded like cardiorespiratory, renal, alcohol or drug related. We present a descriptive cohort study of 28 critically ill patients, who underwent urgent diagnostic laparascopy (UDL) for the investigation of unexplained LA.

## 2 Methods

This was a descriptive cohort study of consecutive 28 patients with unexplained LA. This was a single surgeon's experience over the period of 16 years, from 2001 to 2017. Due to the haemodynamic instability of these patients as determined by the intensivist, they could not be transferred safely to the radiology department for CT scan.

The inclusion cretia was unexplained LA in critically ill patients where extra-abdominal causes were excluded with reasonable confidence and patients were not stable enough to be transferred to radiology department for CT scan.

All patients underwent a standard UDL, which included open Hassan's technique to create CO_2_ pneumoperitoneum via infraumblical incision and two 5 mm ports. A formal diagnostic laparascopy was carried out. The decision whether to proceed with bedside UDL or theatre UDL depended on multitude of factors including but not limited to; the haemodynamic stability of patient, availability of theatre staff, time of day, acceptance of the new concept by the intensive care staff and anaesthetic colleagues and access to emergency theatre. The patients who had positive UDL and required further procedures were transferred to the theatre for remainder of the procedure.

Data was collected by the operating surgeon and analysed retrospectively. Long term outcomes and follow up for these patients were not analysed. Ethical approval was not sought as this was an observational study and diagnostic laparascopy for suspected intra-abdominal sepsis is a routinely used procedure. This work has been reported in line with the STROCSS criteria [11]^.^

## 3 Results

### 3.1 Patient demographics

Twenty eight patients were included in the study (Table 1). Mean age of the patients was 66 (range 43–72) years. There were 8 males and 20 females. Majority of patients were ASA grade 4, (n = 17, 60.7%).

**Table 1:** Patients characteristics.

Patients Characteristics	
Age	66 (43–72) years
Male	8
Female	20

ASA Grade	
1	0
2	0
3	2
4	17
5	9

Referral Route	
ITU	24
Acute Surgical Admission Unit	3
Acute Medical Admission Unit	1

### 3.2 Referral route

Twenty four (85.7%) patients were referred from the intensive therapy unit (ITU), 3 (10.7%) from acute surgical admissions unit and 1 (3.6%) from acute medical admission unit.

### 3.3 Operative time

The mean operating time for UDL was 29 min (range 22–34 min). This does not include the time or any additional procedures, for example proceeding to laparotomy with or without bowel resection.

### 3.4 Findgings of UDL & subsequent treatments

**Fig. 1:**
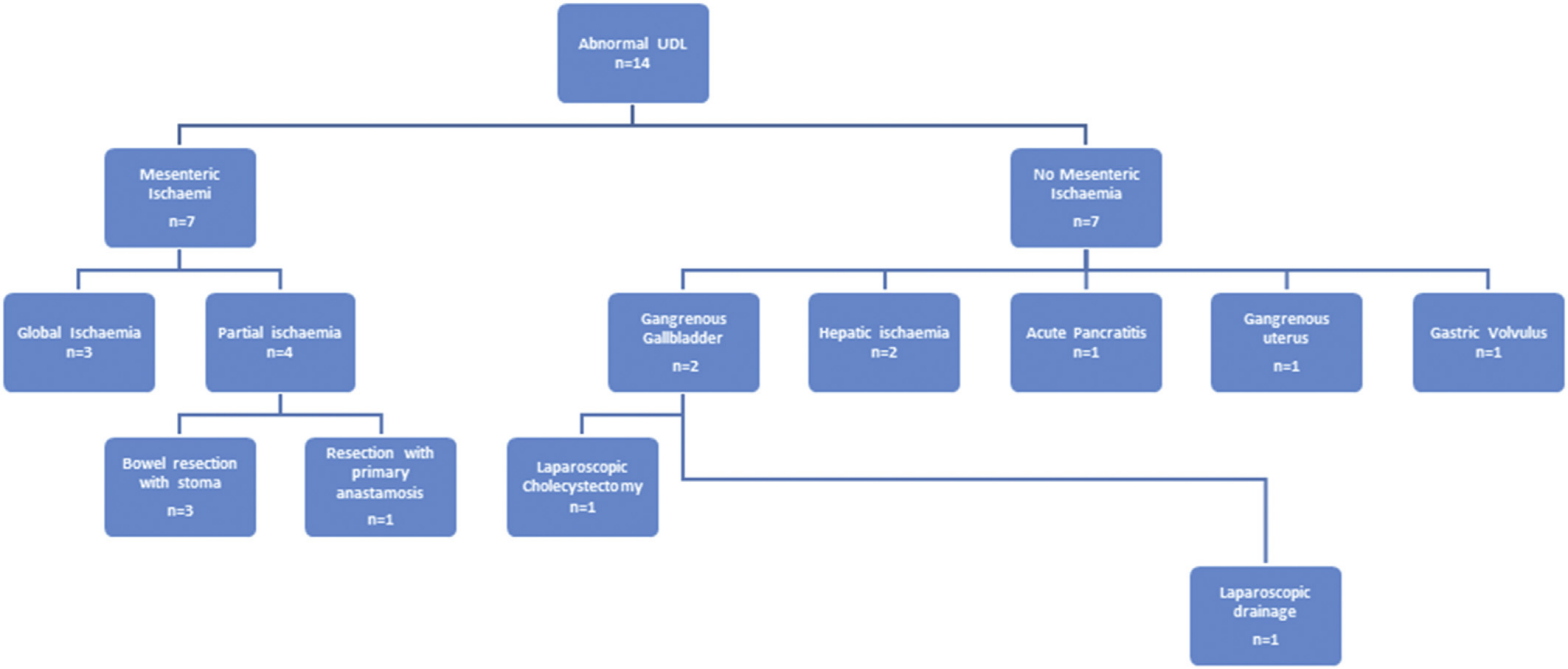
Urgent Diagnostic Laparoscopy (UDL) findings and subsequent procedures.

Fourteen patients (50%) had a normal UDL (Fig. 1); 10 had LA secondary to a low cardiac output state (as determined by invasive monitoring in ITU) and 4 had no identifiable cause. These patients did not require any further surgical intervention.

Fourteen patients (50%) had positive findings on UDL. Seven patients had mesenteric ischaemia; three patients had global ischaemia who were palliated, three patients had bowel resection with stoma formation and one patient had bowel resection with primary anastomosis. Seven patents had no mesenteric ischaemia; two patients had gangrenous gallbladder, one had laparoscopic cholecystectomy and one had laparoscopic drainage. Two patients had hepatic ischaemia. One patient had acute pancreatitis with normal serum amylase, one had gangrenous uterus and one had gastric volvulus with viable stomach requiring laparoscopic gastopexy.

Five patients were converted to laparotomy; 4 for mesenteric ischaemia and 1 for gangrenous uterus. UDL did not contribute to morbidity or mortility in these patients. There was no on-table death and no immediate complications like bleeding, hollow viscus injury and cardiorespiratory complications.

## 4 Discussion

In our case series, an EL was avoided in 23 (82.1%) patients; 14 patients who had a negative diagnostic laparoscopy, 5 with surgically non-salvageable causes and one with normal amylase pancreatitis. The prevailing cause of LA in these patients was a low cardiac output state. A laparotomy was also avoided in patients who had non-salvageable diagnoses, i.e. 3 patients with global mesenteric ischaemia, 2 patients who had hepatic ischaemia. Therefore, the significant morbidity and mortality associated with a negative laparotomy was avoided in over 80% of the patients in this series. UDL correctly identified 8 patients with a surgically treatable cause; 5 of these patients underwent open procedures that were planned based on the findings of the UDL. Other series have also shown that diagnostic laparoscopy is associated with a high negative and positive predictive values [18,20,21].

Intra-abdominal pathology may be the primary cause of sepsis and hence admission to a critical care unit. Common intra-abdominal conditions causing septic shock in the critically ill consist of mesenteric ischaemia, acalculous cholecystitis, pancreatitis, visceral perforation, and intra-abdominal collections [12]. Acalculous cholecystitis is common in these patients due to a combination of prolonged fasting, opioid analgesics, and low cardiac output states [13]. Post-operative mesenteric ischaemia is a recognised complication of aortic surgery [14,15]. Surgical abdomen can occur in up to 5% of neutropenic patients undergoing chemotherapy for haematological malignancies [16]. All of these conditions can lead to a LA due to hypovolemic or septic shock. Any delay in the recognition or management of these conditions can lead to multiple organ dysfunction syndrome and mortality rates that approach 100% [17]. In this subset of patients it is therefore critical that a rapid diagnosis is achieved and definitive intervention proformed.

Critically ill patients are often obtunded, sedated, or anaesthetised, yielding suboptimal information from history and physical examination [18]. Abdominal examination can also be affected by spinal cord injury, a post-operative abdomen and immunocompromised state of a patient [6,16]. Serum investigations can often be non-specific in the critically ill; with leucocytosis, renal impairment and a LA all being relatively frequent findings.

Radiological imaging including ultrasonography (US) and CT scan usually form the next step in diagnosing intra-abdominal pathology. While ultrasonic examination of the abdomen has the advantage of portability, it is mostly utilised for the evaluation of the biliary tree and is less useful in the presence of gaseous distension of bowel. It is also not likely to be diagnostic in cases of mesenteric ischaemia. Diagnostic yield from US examination of the abdomen is also operator dependant [[19], [20], [21]]. It is now common to utilise CT scanning to evaluate potential abdominal cause of LA in critically ill patients. The accuracy of CT in critically ill patients varies between 78% and 89% and can be non-specific in subtle cases of mesenteric ischaemia [8,20,22]. CT also has the disadvantage of requiring the critically ill patient to be transferred to the radiology suite [23]. The process of transferring a critically ill patient for radiological investigations or to the theatre suite can be fraught with complications, including hypotension, respiratory distress, line disconnections, and cardiac dysrhythmia [[24], [25], [26], [27], [28]].

This often leaves the general surgeon with a conundrum; a critically ill patient with an unexplained LA, a possible intra-abdominal cause and with no definitive diagnosis by non-invasive methods. Delaying a diagnosis and definitive management at this juncture is associated with a mortality rate that approaches 100% [8,28,29]. This can often result in EL being utilised as both a diagnostic and therapeutic modality. However, a laparotomy alone can be associated with a morbidity rate that varies from 5% to 22% [9]. There is also no guarantee that the risk of morbidity will be balanced by the benefits of a negative laparotomy as some series have noted a 90% mortality rate with a negative laparotomy in critically ill patients; with the caveat that it would be difficult to isolate the contribution of a negative laparotomy to the mortality in this subset of patients [10,30,31]. Laparotomy is also associated with the added potential problems of wound complications, dehiscence, prolonged ileus, fluid shifts, and iatrogenic visceral perforation [20].

With the advent of minimally invasive surgery, the role of laparoscopy to evaluate intra abdominal pathology has increased in critical care. But still there is reservation and low uptake of UDL in criticle care unit. For abdominal sepsis for example, there were preliminary concerns over its effect on haemodynamic compromise of patients. Using porcine sepsis and shock models, it was shown that animals that were exposed to laparoscopy expressed substantial hypercarbia and diminished cardiac index. However, further studies showed that aggressive fluid resuscitation partially ameliorated these effects. Several studies have successfully shown the efficacy of this technique in critically ill patients. Cerribelli et al., conducted a retrospective study on 62 patients who underwent bedside laparoscopy for an acute abdomen. Their results showed that laparoscopy proved a safe procedure, as haemodynamic alterations were minimal, diagnostic accuracy was high and laparotomy was prevented [3]. There are other studies that have observed similar findings [[4], [5], [6]].

We proposed that UDL at the bedside or in the operating suite is a valid investigation in the critically ill with an unexplained LA. UDL allows the surgeon to directly visualise the peritoneal cavity and where required to carry out a therapeutic intervention. The equipment required for UDL is readily available in most theatre suites utilising a stacking system where the monitor, video unit, light source, and insufflator are on a mobile cart. The size of the incisions are small with minimal or no exposure of the intra-abdominal contents, allowing the procedure to be carried out at the bedside in ITU if necessary.

The therapeutic potential of laparoscopy in unexplained LA was also demonstrated in our case series. Two patients with acalculous gengenous cholecystitis and another with a gastric volvulus underwent laparoscopic cholecystectomy and gastropexy respectively. Four patients with localised mesenteric ischaemia and one patient with a gangrenous uterus had laparotomies in the theatre suite.

Our experience has shown that the attitude of the ITU staff was apprehensive towards laparoscopy in these critically ill patients in ITU settings. Much of this, we suspect, is due to the lack of education about this relatively benign nature of this procedure. As laparoscopy is conventionally undertaken in a theatre setting, this technique can be safely transferred to bedside setting without much alteration in patient haemodynamics. No extra resourses are required from ITU personel. But on the other hand the theatre staff has to bring the equipment to the bed side. To handle this procedure are not more than what was required for other procedures done in ITU i.e. colonoscopy, gastroscopy, bronchoscopy and tracheostomy etc.

This study was limited by the sample size and single surgeon experience. We were also limited by a lack of definitive diagnoses in those with a negative diagnostic laparoscopy. For patients who have died, post-mortem examinations would have been helpful but consent was unobtainable for a variety of reasons. As the study period extends more than one decade, the surgical practise has slowly changed as laparoscopy is becoming more common. There is no long term follow up for the patients, however the aim of the study was to look at the outcome related to this diagnostic tool. Also, as the CT scan are becoming more common with better diagnostic yeild and radiology department a is considered a safer place than historically thought, as a consequence the intensivists are now taking much sicker patients to the radiology departments than ever before.

## 5 Conclusion

UDL is a feasible, accurate and most importantly, safe modality for the investigation of LA the critically ill patient. It removes the need for an EL in the high risk patient and reduces the risks associated with patient transfer if performed at the bedside. UDL would also be a useful adjunct when converting to an open procedure; particularly when planning the primary incision. It does not require a large investment in terms of equipment or expertise and the relatively short length of the procedure coupled with the invasive monitoring likely already in place makes it less likely to negatively impact on the haemodynamic parameters of the patient. Although it is far from being widely accepted, bedside UDL should be included in the diagnostic algorithm when evaluating LA in the critically ill.

## Ethical approval

We did need ethical approval for this study in our institution as laparoscopy is well known option for treatment of patients such included in our study.

## Sources of funding

None.

## Author contribution

First Author M A Sajid: Study design, data analysis, writing, data collection.

Second Author K S Khan: Data collection, writing.

Corresponding Author: Data Analysis.

## Conflicts of interest

None.

## Trial registry number

N/A.

## Guarantor

Zulfiqar Hanif-corresponding Author.

## Research registration unique identifying number (UIN)

Research Registry 4100.

## Disclosure

All authors have no conflicts of interest or financial ties to disclose.

## Provenance and peer review

Not commissioned, externally peer reviewed.

## Appendix A Supplementary data

Authr Form ZH Authr Form ZH
